# Adaptive nodes enrich nonlinear cooperative learning beyond traditional adaptation by links

**DOI:** 10.1038/s41598-018-23471-7

**Published:** 2018-03-23

**Authors:** Shira Sardi, Roni Vardi, Amir Goldental, Anton Sheinin, Herut Uzan, Ido Kanter

**Affiliations:** 1 Department of Physics, Bar-Ilan University, Ramat-Gan, 52900 Israel; 2 Gonda Interdisciplinary Brain Research Center and the Goodman Faculty of Life Sciences, Bar-Ilan University, Ramat-Gan, 52900 Israel; 3 Sagol School of Neuroscience, Tel Aviv University, Tel Aviv, Israel

## Abstract

Physical models typically assume time-independent interactions, whereas neural networks and machine learning incorporate interactions that function as adjustable parameters. Here we demonstrate a new type of abundant cooperative nonlinear dynamics where learning is attributed solely to the nodes, instead of the network links which their number is significantly larger. The nodal, neuronal, fast adaptation follows its relative anisotropic (dendritic) input timings, as indicated experimentally, similarly to the slow learning mechanism currently attributed to the links, synapses. It represents a non-local learning rule, where effectively many incoming links to a node concurrently undergo the same adaptation. The network dynamics is now counterintuitively governed by the weak links, which previously were assumed to be insignificant. This cooperative nonlinear dynamic adaptation presents a self-controlled mechanism to prevent divergence or vanishing of the learning parameters, as opposed to learning by links, and also supports self-oscillations of the effective learning parameters. It hints on a hierarchical computational complexity of nodes, following their number of anisotropic inputs and opens new horizons for advanced deep learning algorithms and artificial intelligence based applications, as well as a new mechanism for enhanced and fast learning by neural networks.

## Introduction

**Figure 1:**
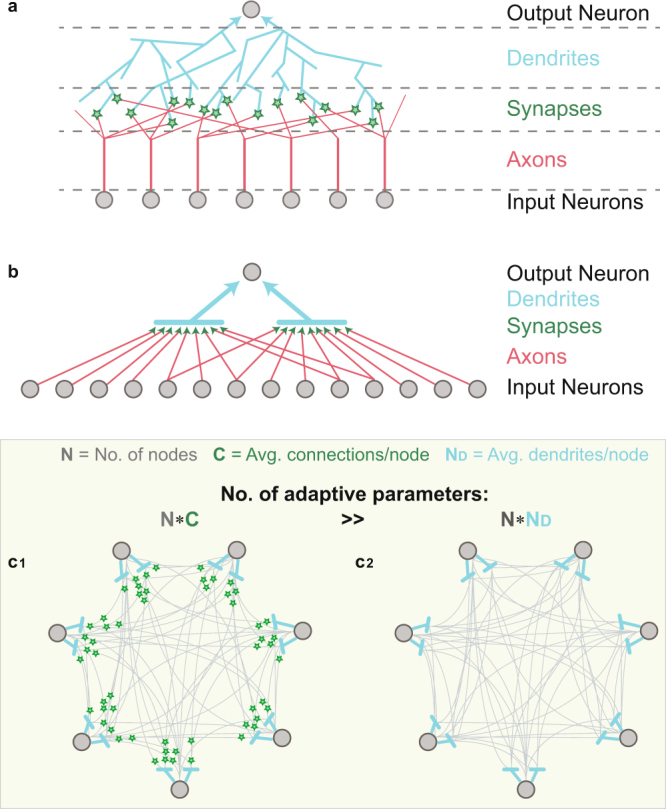
From biological details to a schematic diagram representing the traditional learning by links (synapses) and the proposed learning by nodes. (**a**) A biological schema of an output neuron composed of a soma (gray circle, top) with two roots of dendritic trees (light-blue arrows), splitting into many dendritic branches (light-blue lines). The signal arriving from each of the connecting input neurons (gray circles, bottom) travels via its axon and its many branches (red lines) until terminating at the meeting points with the dendrites, the synapses (green stars), where some branches travel to other neurons. (**b**) A simplified schema of **a** where a dendritic tree and its branches are denoted by a horizontal light-blue line and synapses by green arrows. (**c**) A fully connected network composed of N = 7 nodes (gray circles) with 2 dendritic trees per node (light-blue). (**c**_**1**_) Each node receives inputs from all other nodes via synapses (green stars). The number of adaptive parameters, synapses, is N*C, where C is the average input connectivity (O(N^2^) in dense networks). (**c**_**2**_) Similar topology to **c**_**1**_, where the number of adaptive parameters is only O(N), the number of dendritic trees, N*N_D_, where N_D_ is the average number of dendritic trees per node.

Research in neurophysiology reveals detailed structures of neural networks, including a large diversity among their building blocks. The coarse picture is a unidirectional network, where each neuron collects the incoming signals from its input neurons (Fig. 1a). Specifically, the output neuron collects its incoming inputs via several dendritic trees^1^ (light-blue lines in Fig. 1a), where each input neuron transmits its output signal via a single axon (red lines in Fig. 1a). The connections between the many branches of the axon and the dendritic trees are called synapses (green-stars in Fig. 1a), which bridge between the output and the input signals. The current assumption is that synapses slowly change their strengths during the learning process^2–5^. A simplified scheme is demonstrated in Fig. 1b, where the branches of each neuronal dendritic tree are represented by a bar (light-blue), with multiple connecting synapses (green-arrows), where each input neuron (gray) can be connected to several dendritic trees, a scheme which is also used in deep learning^6–8^.

A network of connecting neurons consists of *N* × *C* learning parameters (synapses), where *N* and *C* stand for the number of nodes and the average incoming connections per node, respectively (Fig. 1c_1_). In the approach suggested here, the network consists of *N* × *N*_*D*_ learning parameters, which are the strengths of the dendritic trees, where *N*_*D*_ is the average number of dendritic trees per neuron (Fig. 1c_2_). The parameter *C* scales as O(N) in fully connected or dense networks and is estimated^9^ in neural networks to be O(10^4^). This number is significantly larger than *N*_*D*_, since typically there are only a few dendritic trees per neuron^9–11^.

Our central objective is to compare the cooperative dynamical properties between synaptic (link) and dendritic (nodal) learning scenarios (Fig. 1c). In particular, we examine whether the attribution of the learning process to much fewer adjustable parameters, *N* × *N*_*D*_, enriches or diminishes the learning capabilities. Finally, the consensus that the learning process is attributed solely to the synapses is questioned. A new type of experiments strongly indicates that a faster and enhanced learning process occurs in the neuronal dendrites, similarly to what is currently attributed to the synapses^3,11^.

## Results

### Two dendrites

**Figure 2:**
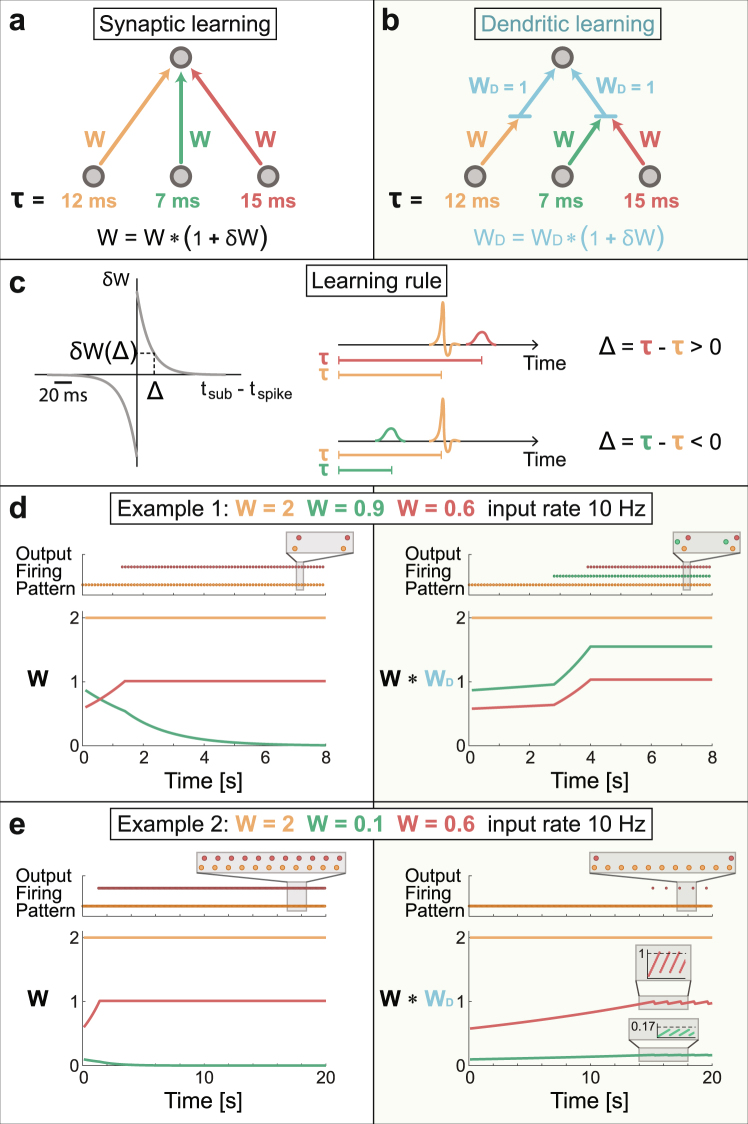
Different stationary firing patterns and weights for learning by links and nodes in a feedforward network. (**a**) A schema of a perceptron with three input units, connected to one output unit with weights and delays (w, τ, color coded). The relative change in the strength of a weight during a learning step is δW (defined in **c**). The dynamics of each unit is governed by leaky integrated-and-fire neuron (Methods). (**b**) The same perceptron and delays as in **a** but the first input is connected to the output node via the left-dendrite, while the two other inputs are connected via the right-dendrite. The dendritic weights and their relative changes during a learning step are denoted by W_D_ and δW, respectively. The initial weights for both dendrites are W_D_ = 1. (**c**) Left: A typical profile for δW = 0.05*exp(−|Δ|/15)*sign(Δ) during a learning step, where Δ stands for the time-lag between a spike and a sub-threshold stimulation, measured in ms. Right: Scenarios for positive/negative Δ, spikes colored in orange and sub-threshold stimulations are denoted by (green and red) hills. (**d**) An example of the initial three weights (color coded), where the input units are simultaneously stimulated at 10 Hz. Left: The dynamical evolution of the three weights in **a** (bottom) and the firing timings of the output unit (dots at upper part), colored following the origin of the above-threshold stimulation. Right: Results for **b** where initially W_D_ = 1. (**e**) Similar to **d** but with different initial weights. The stationary firing patterns in **d** and **e** are the same for synaptic learning in **a**, but differ for dendritic learning in **b**.

A comparison between the two types of learning processes is first examined using a prototypical feedforward network, a perceptron^12–14^ consisting of three input nodes, one output node and three weights with given delays and initial strengths (Fig. 2a). For synaptic learning, the adjustable parameters are the three weight strengths (color coded in Fig. 2a). For dendritic learning the weight strengths (W) are unchanged and the adjustable parameters are the two dendritic strengths (W_D_ in Fig. 2b), connected to the first and to the last two input units, respectively (Fig. 2b). The synaptic and dendritic adaptations are identical and are based on the currently accepted modified Hebbian learning rule, known as spike-time-dependent-plasticity^3,4,11^. Specifically, the relative change in the strength of a weight, δW, during a learning step is a function of the time-lag, Δ, between an above-threshold stimulation, resulting in an evoked spike, and a stimulation that does not result in an evoke spike, e.g. sub-threshold stimulation. A positive/negative Δ strengthens/weakens a weight following a typical profile (Fig. 2c).

The input units are stimulated above threshold simultaneously at 10 Hz and the following standard leaky integrate-and-fire model^15,16^ is used to evaluate the dynamics of the output neuron for both scenarios (Fig. 2a,b). Specifically, we simulated a perceptron consisting of N = 3 excitatory leaky integrate and fire input neurons and one output neuron. The voltage V(t) of the output neuron is given by the equation:

1 $$\frac{dV}{dt}=-\frac{V-{V}_{st}}{\tau }+\sum _{i=1}^{N}({W}_{i}\ast {W}_{Di})\sum _{n}\delta (t-{t}_{i}(n)-{d}_{i})$$

where W_i_ and W_Di_ are the connection’s and dendrite’s strength from neuron i to the output neuron, respectively. d_i_ is the delay from neuron i to the output neuron, and N stands for the number of input neurons. τ = 20 ms is the membrane time constant and V_st_ = −70 mV stands for the stable membrane (resting) potential. The summation over t_i_(n) sums all the firing times of neuron i. A neuronal threshold is defined as V_th_ = −54 mV and a threshold crossing results in an evoked spike. In the event of synaptic learning, for every pair of a sub-threshold stimulation and an evoked spike, the weights, W_i_, were modulated according to the learning curve (Fig. 2c). Similarly, in the event of dendritic learning, for every pair of a sub-threshold stimulation and an evoked spike, originated from two different dendrites, the dendritic weights, W_Di_, were modulated following the learning curve (Fig. 2c, see Methods for more details).

For synaptic learning (Fig. 2d, left), evoked spikes are initially generated by the orange-weight, and the preceding/later sub-threshold stimulation weakens/strengthens the green/red weight, respectively (Fig. 2c). Asymptotically, the green-weight vanishes and the red-weight is at threshold and the perceptron repeatedly generates pairs of evoked spikes (Fig. 2d, left).

For dendritic learning (Fig. 2d, right), an evoked spike is generated by the left dendrite after 12 ms (orange) and two sub-threshold stimulations arrive via the right dendrite after 7 ms (green) and 15 ms (red), 5 ms before and 3 ms after an evoked spike, respectively. Consequently, the right dendrite is strengthening on the average (Fig. 2c). Asymptotically, all three effective weights, W*W_D_, are above-threshold and generate triplets of evoked spikes (Fig. 2d, right).

The same perceptron but with different initial weights results for synaptic learning in the same firing pattern and weight strengths (Fig. 2d,e, left). For dendritic learning (Fig. 2e, right), the right-dendrite strengthens such that the red-weight is effectively above-threshold, while the green-weight is still sub-threshold (Fig. 2e, right at ~15 s). The firing patterns consist now of orange-red pairs of spikes (Fig. 2e, right), however the learning process proceeds. The green sub-threshold stimulation arrives before the orange-spike, resulting in the weakening of the right-dendrite and the termination of red-spikes. Now the right-dendrite is again strengthening as in Fig. 2d and so forth, resulting in a complex firing pattern with a longer periodicity.

Examples presented in Fig. 2d,e hint on two major differences between the two learning scenarios. For the same architecture but different initial weights, synaptic learning tends to stabilize on the same firing pattern, whereas dendritic leaning may result in a variety of firing patterns. In addition, synaptic learning drives weights to extreme limits^17,18^, vanishing or threshold, whereas dendritic learning enables stabilization around intermediate values.

### Three dendrites

**Figure 3:**
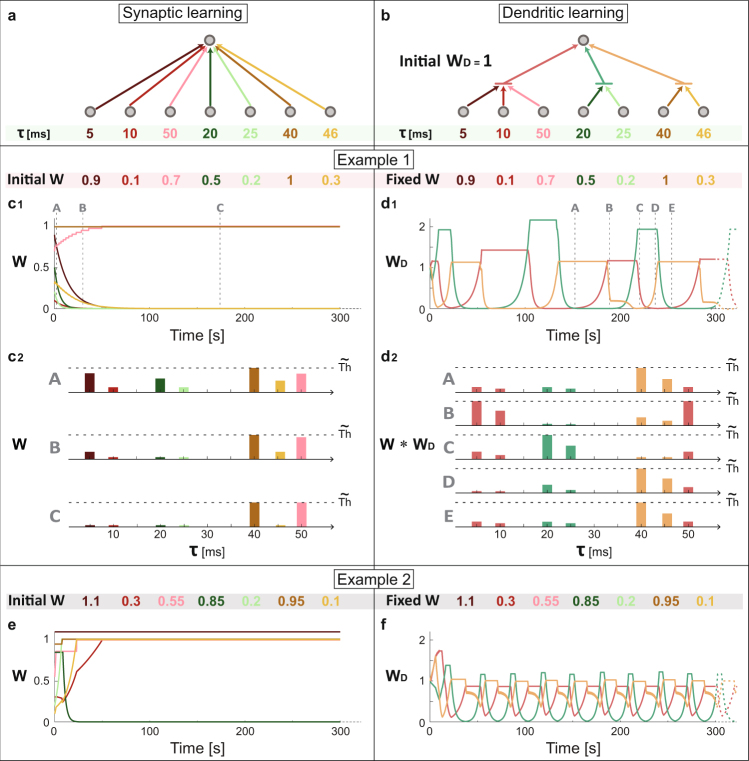
Dendritic learning as a self-controlled mechanism for oscillating weights, governed by the weak links. (**a**) A schema of a perceptron with seven inputs with weights and delays (w, τ, color coded). Changes in weights during the learning are defined in Fig. 2c, and the dynamics of the output is governed by leaky integrated-and-fire neuron (Methods). (**b**) A similar perceptron and delays as in **a**, but the output unit has three dendrites (color coded), red/green/orange connecting 3/2/2 input units, respectively, and with given initial dendritic weights, W_D_. (**c**_**1**_) The initial seven weights in **a** are denoted (top, color coded). The input units are simultaneously stimulated at 10 Hz and the resulting dynamical evolution of the seven adaptive weights is presented. (**c**_**2**_) Schematic presentation of the seven weights, ordered following their delays, with respect to $\widetilde{{\rm{Th}}}$, the difference between the threshold and the current neuronal voltage, at three denoted timings (A–C) in **c**_**1**_. (**d**_**1**_) Similar to **c**_**1**_ where the same initial seven weights are now time-independent and the three dendritic weights in **b** are dynamically updated. (**d**_**2**_), Similar to **c**_**2**_ where the seven effective weights W*W_D_ (color coded following their dendrites and ordered following their delays) are presented at five denoted timings (A–E) in **d**_**1**_. (**e**) and (**f**) present similar results as in **c**_**1**_ and **d**_**1**_ respectively, for a different set of seven initial weights.

An extension to a perceptron with seven inputs (N = 7 in the abovementioned equation) and with three dendrites enriches the fundamental differences between the two adaptive dynamics (Fig. 3a,b). The seven delays (Fig. 3a,b, bottom) and initial weights (Fig. 3c_1_ and d_1_, top) are identical for both scenarios and the input units are simultaneously stimulated above-threshold at 10 Hz. Weights in synaptic learning are driven again toward vanishing or threshold limits (Figs 3c_1_ and 2d,e), however, dendritic learning reveals a new phenomenon, oscillatory behavior of the weights. These trends are explained using several snapshots of the effective weights, color coded and ordered following their delays, representing different stages of the dynamics (Fig. 3c_2_ and d_2_). Since the neuronal voltage has a decay time to the resting potential after an input arrival (Methods), a necessary condition to generate an evoked spike is an effective weight which reaches $\widetilde{{\rm{Th}}}$, the difference between the threshold and the current neuronal voltage. For synaptic learning, initially only the dark-orange weight is at threshold (panel A in Fig. 3c_2_). Following the learning rule (Fig. 2c), the strengths of all longer/shorter delays increase/decrease (panel B in Fig. 3c_2_), until only vanishing or weights at threshold remain (panel C in Fig. 3c_2_).

For dendritic learning, initially only the effective orange weight (W*W_D_) is at threshold (panel A in Fig. 3d_2_), generating a spike 40 ms after each input stimulation. Consequently, the red-dendrite and effectively its three incoming weights are strengthening (panel B in Fig. 3d_2_), since its nearby sub-threshold input, via the 50 ms pink-weight, arrives 10 ms later (Fig. 3b). Similarly, the strength of the green-dendrite decreases as it generates sub-threshold stimulations prior to the evoked spikes. Spikes are now generated after 5 ms and also after 50 ms (panel B in Fig. 3d_2_) and the strength of the orange-dendrite rapidly decreases, since its sub-threshold input arrives just before, after 46 ms (The origin of the orange decay-slope shape is demonstrated in Fig. S1). The red-evoked spike at 5 ms is now rapidly strengthening the green-dendrite (with 20 ms and 25 ms delays) until generating evoked spikes (panels B and C in Fig. 3d_2_). The 10 ms red-weight sub-threshold stimulations, arriving before the green-spikes, weaken the red dendrite and the red-spikes terminate (panel C in Fig. 3d_2_). In addition, the orange-dendrite is strengthening and finally generates evoked spikes, as its sub-threshold stimulations arrive after the green-spikes (panel D in Fig. 3d_2_). Now green-spikes terminate, as a result of green-sub-threshold stimulation at 25 ms, prior to the orange-spikes (panels D,E in Fig. 3d_2_). A loop of the weight strengths emerges (panels E and A in Fig. 3d_2_) generating an oscillatory behavior (Fig. 3d_1_). Identical architectures (Fig. 3a,b) but with different initial weights (Fig. 3e,f) result again in extreme limit weights for synaptic learning, but with a different oscillatory behavior for dendritic learning.

Synaptic learning terminates in vanishing or threshold weights, independent of the initial conditions (Figs 2 and 3) and represents an unrealistic biological reality. In addition, the large fraction of very weak weights has practically no impact at all on the dynamics. In contrast, dendritic learning can stabilize weights with intermediate strengths (Fig. 2e, right) and oscillatory behaviors (Fig. 3d_1_,f) which are significantly and instantaneously governed by the sub-threshold stimulations originated from the weak effective couplings.

### Experimental results

**Figure 4:**
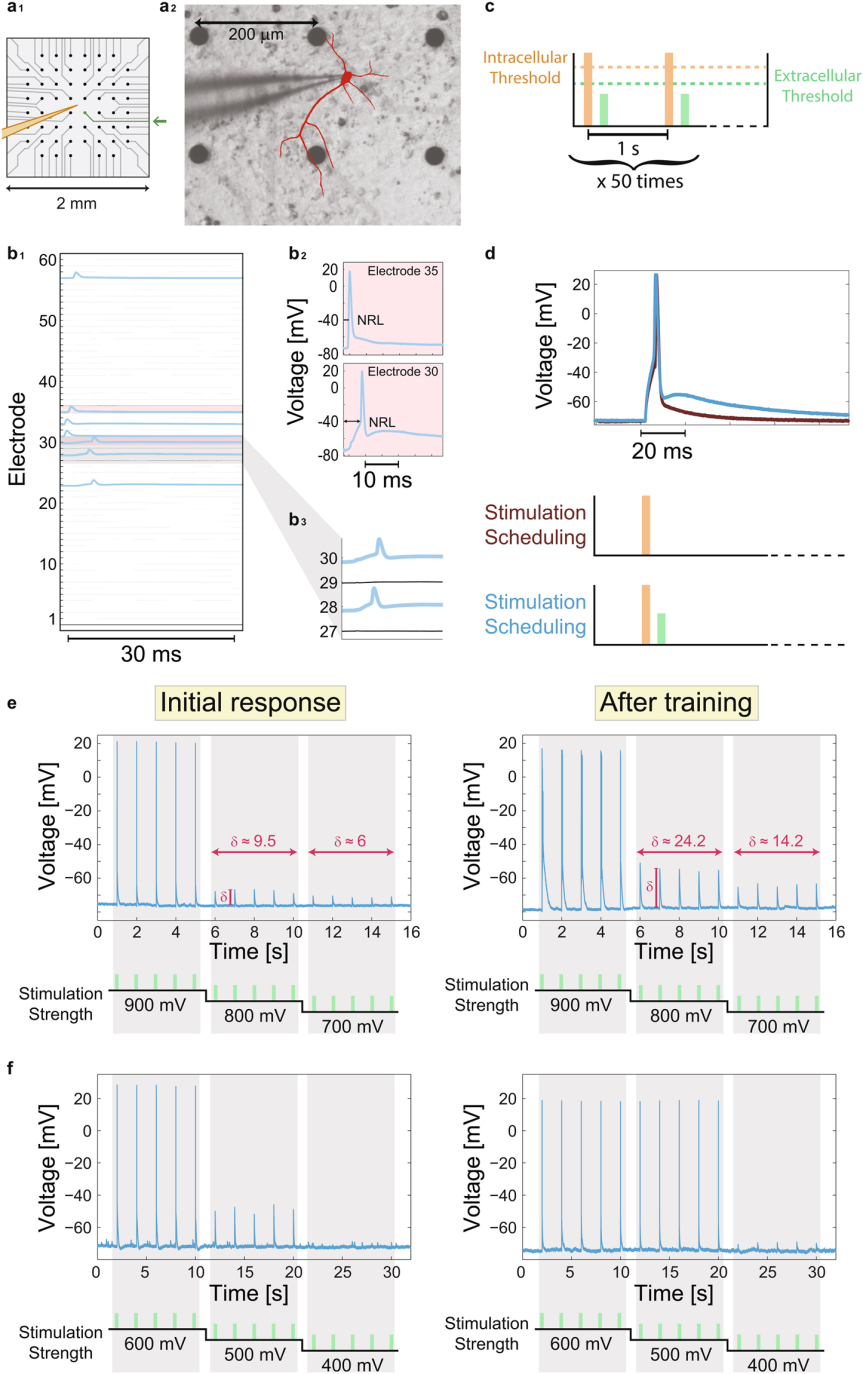
Experimental results indicating enhanced dendritic learning following the relative timings of the neuronal anisotropic inputs, similar to the mechanism currently attributed to links. (**a**_**1**_) A zoom-in of a micro-electrode array (MEA) consisting of 60 extracellular electrodes separated by 200 µm, indicating a patched neuron by an intracellular electrode (orange) and a nearby extracellular electrode (green). (**a**_**2**_) A patched neuron and its dendrites (red), growing to different directions and in proximity to extracellular electrodes are presented using reconstruction of a fluorescence image (Methods). (**b**_**1**_) A procedure for finding reliable evoked spikes by a subset of extracellular electrodes. The 60 electrodes are stimulated serially several times at 2 Hz (presented here once) and the recorded intracellular voltage (blue in case of evoked spike) is presented (Methods). (**b**_**2**_) A zoom-in of the pink area in **b**_**1**_, presenting evoked spikes originated from different extracellular stimulating electrodes and the NRL. (**b**_**3**_) A zoom-in of 4 electrodes from **b**_**1**_, showing electrodes with/without recorded evoked spikes. (**c**) A typical stimulation scheduling of the learning process of an above-threshold intracellular stimulation (orange) and a sub-threshold extracellular stimulation (green), separated by a fixed time delay, 2–5 ms (Methods), and such pairs are given 50 times at 1 Hz. (**d**) A comparison between the spike waveform resulted from an intracellular stimulation (red) where the same intracellular stimulation is followed by an adjacent (scheduling at the bottom) extracellular sub-threshold stimulations (light-blue). (**e**) An intracellular voltage recording of a patched neuron stimulated extracellularly 5 times at 1 Hz at each noted stimulation amplitude (bottom). Left: Initial response before training. Right: Several minutes after training (Methods). δ measures the height of the voltage peak (local depolarization), averaged over 5 stimulations with a given amplitude, in comparison to the resting potential, indicting an enhancement of δ by 200–300% by learning. (**f**) Similar to **e** where stimulations were given at 0.5 Hz. The effect of the learning is expressed by the appearance of spikes after training instead of small depolarization before (at 500 mV).

The reality of the theoretical concept of dendritic learning receives a support from the following new type of *in-vitro* experiments, where synaptic blockers are added to neuronal cultures such that sparse synaptic connectivity is excluded (Methods). A multi-electrode array (Fig. S2) is used to stimulate extracellularly a patched neuron^19^ via its dendrites (Fig. 4a). An online method is used to identify a subset of extracellular electrodes which reliably generate intracellularly evoked spikes (Fig. 4b). Low stimulation rates (e.g. 1 Hz) ensure stable neuronal response latencies, NRL, measuring the time-lag between the extracellular stimulation and the intracellularly recorded evoked spike, which is crucial for controlling the relative timings between pairs of intra- and extra- stimulations^20^.

The learning process is based on a training set of typically 50 pairs of stimulations, an above-threshold intracellular stimulation followed by an extracellular stimulation which does not result in evoke spikes, e.g. sub-threshold (Fig. 4c), arriving after a predefined delay, typically 2–5 ms to enhance possible adaptation (Fig. 2c, Methods). We take into account only experimental realizations where a local depolarization was visible by a consecutive sub-threshold stimulation to the above-threshold one (Fig. 4d). The demonstrated results were quantitatively repeated tens of times on many cultures (see statistical analysis in Methods).

The intracellular voltage recordings of a patched neuron stimulated extracellularly before and a few minutes after training (Fig. 4c) presents a significant effect of the learning in the form of 200–300% increase in the local depolarization (Fig. 4e). This learning effect emerges only a few minutes after the termination of the training procedure and was found to be stable and persistent over longer periods (by repeated measurements of solely extracellular stimulations over tens of minutes). Another evidence for such learning is the enhancement of the effect of extracellular stimulations from small local depolarization to evoked spikes recorded intracellularly (Fig. 4f). Note that before training, the responsiveness of neurons was found to be time-independent and over tens of minutes.

**Figure 5:**
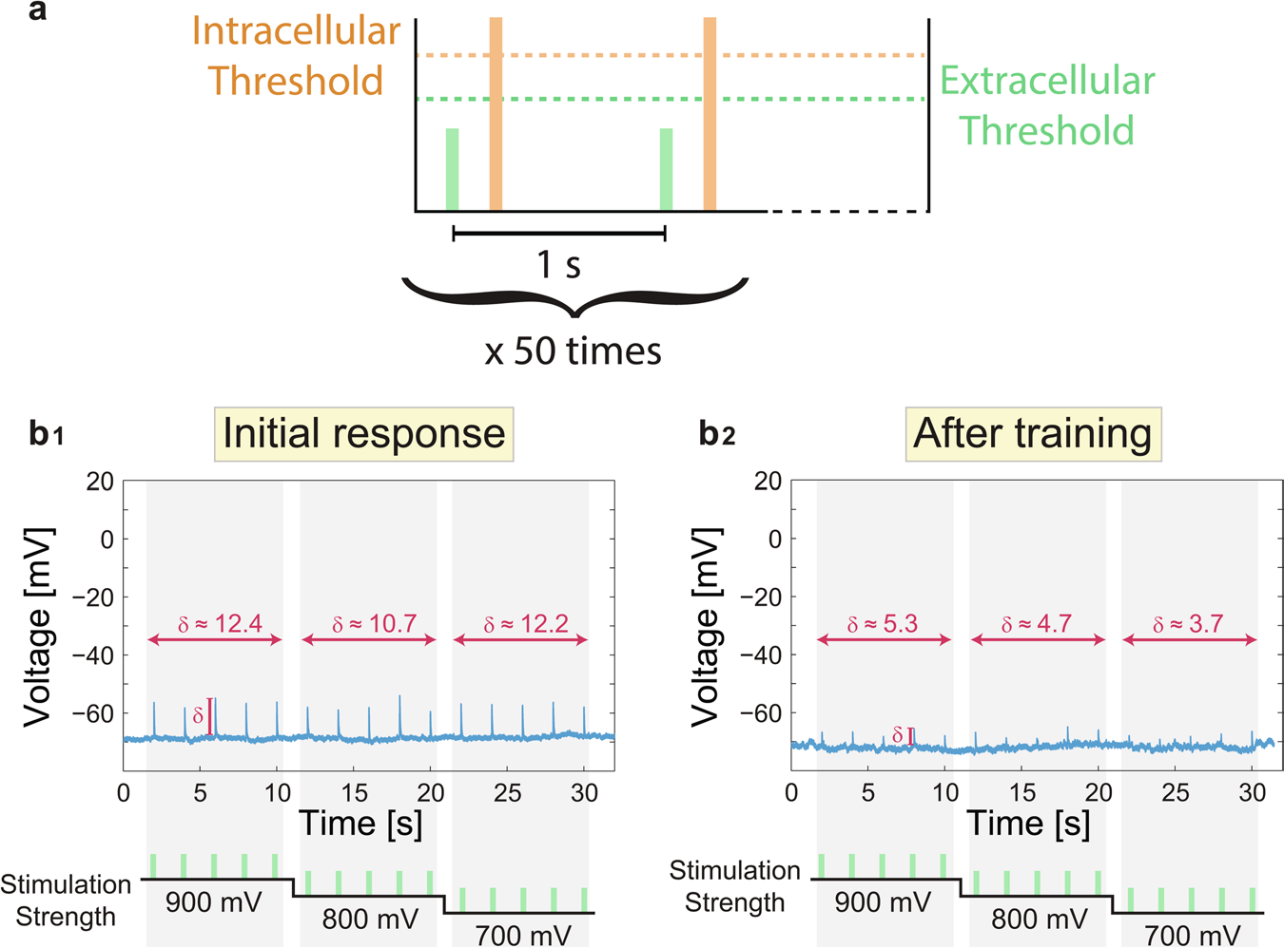
Experimental results indicating reverse dendritic learning: sub-threshold extracellular stimulation is given 5 ms prior to the above-threshold intracellular one. (**a**) A typical stimulation scheduling of the learning process, of a sub-threshold extracellular stimulation (green) and an above-threshold intracellular stimulation (orange), separated by a fixed time delay, 5 ms, and such pairs are given 50 times at 1 Hz. (**b**) An intracellular voltage recording of a patched neuron stimulated extracellularly 5 times at 0.5 Hz at each noted stimulation amplitude (bottom). (**b**_**1**_) Initial responses before training. (**b**_**2**_) Several minutes after training (Methods). δ measures the height of the voltage peak (local depolarization), averaged over 5 stimulations with a given amplitude, in comparison to the resting potential. Results indicate a decrease of δ by 55–70% by reverse learning.

A reverse learning procedure, presenting the sub-threshold stimulation prior to the above-threshold one, was also examined in tens of experiments, indicating no effect or weakening of the local depolarization, but no strengthening (Fig. 5). It suggests, as indicated by some preliminary results, the possibility to first strengthen and then weaken the local depolarization, using sequential learning and reverse learning.

## Discussion

Most of the neural network links have relatively weak strengths in comparison to the threshold^21,22^. Hence, a persistent cooperation among many stimulation timings is required to reliably influence the dynamics, otherwise most of the links are actually dynamically insignificant. Using a nodal (dendritic) learning rule, we show that the dynamics is counterintuitively mainly governed by the weak links (Figs 2 and 3). Interestingly, the nodal learning exhibits a self-controlled mechanism for achieving intermediate and oscillatory weight strengths, as opposed to learning by the links, and hints on new horizons for online learning^23^. The emergence of fast (Fig. 2e) and slow (Fig. 3) oscillations as a result of the learning process might be related to high cognitive functionalities and a source for transitory binding activities among macroscopic cortical regions^24^. These oscillations were found to be robust also to the anisotropic nature of neurons^25,26^ and have to be distinguished from oscillations emerging from the stochastic neuronal responses^20^. The presented nodal adaptation questions the objective of the similar accepted slower learning rules of tens of minutes by the links, which are probably done in a serial manner (Fig. 1).

The experimental results were obtained using solely cortical pyramidal neurons (Methods), and call to examine their generality using other types of neurons. In addition, the experiments were designed such that the sub-threshold stimulation arrives shortly after or before the spike (2–5 ms) in order to enhance the effect of adaptation. To recover the full learning curve (Fig. 2c), more detailed experiments are required.

The adaptation process was examined when an extracellular sub-threshold stimulation was given after or before an intracellular above-threshold stimulation. Preliminary results indicate that a similar adaptation occurs also in the scenario of solely two sources of extracellular stimulations, one above- and one sub-threshold. The time-lag between the arrivals of both stimulations to the neuron was tuned carefully, taking into account the NRL, in order to imitate a similar scenario to Fig. 4e,f. Preliminary results also indicate the possibility to strengthen and then weaken the local depolarization by consecutive nodal learning and reverse nodal learning (Figs 4 and 5). The observation of the oscillatory behavior of the strength of a dendrite is a necessary condition to verify the similarity between the theoretical predictions (Figs 2 and 3) and experimental observations. It requires a stable control over intra- and extra- stimulations of several patched neurons which constitute small networks (Figs 2 and 3), which is currently beyond our experimental capabilities.

The oscillatory behavior is exemplified for a few specific sets of weights and delays (Figs 2 and 3), however, it represents a generic behavior. The architectures of a neuron with two or three dendrites, where each dendrite has several synapses, were simulated for a few thousands of sets of initial conditions, i.e. synaptic delays and synaptic weights. Specifically, synaptic delays were randomly chosen between 1 and 50 ms, with a gap of at least 3 ms between synaptic delays belonging the same dendrite, and with the constraint that the minimal and the maximal synaptic delays belong to the first dendrite (as in Figs 2 and 3). Weights were randomly chosen from a uniform distribution between 0.1 and 1.8, and at least one effective weight is above threshold, in order to initiate firing. Results indicate that a large fraction of random initial conditions leads to oscillatory behaviors, e.g. ~0.53 for three dendrites with three synapses per dendrite.

The slow oscillatory behavior of the effective weight strengths is realized using a node with three adaptive dendrites, but is unreachable in our scheme using a node with two adaptive dendrites. It hints on a computational hierarchical among networks following the complex morphology of their nodes, e.g. the number of their dendrites^27,28^. In addition, preliminary results indicate that the different number of firing patterns, stationary or oscillatory, obtained for the nodal learning can exceed dozens in scenarios of only a few adjustable parameters, dendrites. More precisely, for a given architecture and delays, the number of different firing patterns is estimated using different initial time-independent synaptic weights, and is found to exceed a hundred, for instance, for feedforward networks consisting of three adjustable dendrites. These manifolds of firing patterns might be relevant to realities where each dendrite has many input synapses but only a subset is deliberately activated. In addition, preliminary results indicate that one can find several firing patterns for given synaptic strengths and different initial conditions for the dendritic strengths. The large number of time-dependent firing patterns, compared to the number of adjustable parameters, indicates that notions like capacity of a network, capacity per weight and generalization have to be redefined in the light of nodal learning. Results call to examine the features of such dynamics, including the possible number of oscillatory attractors for the weights, using more complex feedforward and recurrent networks and their implication on advanced deep learning algorithms^29–34^.

## Methods

### Simulations

#### Methods of Simulation

We simulated a perceptron consisting of N = 3 (Fig. 2) and N = 7 (Fig. 3) excitatory leaky integrate and fire input neurons and one output neuron using equation 1, where W_i_ and W_Di_ are the connection’s and dendrite’s strength from neuron i to the output neuron, respectively. d_i_ is the delay from neuron i to the output neuron and N stands for the number of input neurons. τ = 20 ms is the membrane time constant and V_st_ = −70 mV stands for the stable membrane (resting) potential. The summation over t_i_(n) sums all the firing times of neuron i. A neuronal threshold is defined at V_th_ = −54 mV and a threshold crossing results in an evoked spike followed by a refractory period of 2 ms. During the refractory period no evoked spikes are possible and the voltage is set to V_st_. For simplicity, we scale the equation such that V_th_ = 1, V_st_ = 0, consequently, V > = 1 is above threshold and V < 1 is below threshold. Nevertheless, results remain the same for both the scaled and unscaled equation. The initial voltage is V (t = 0) = 0 and w_Di_ = 1.

#### Connectivity

The connectivity was designed as stated for each experiment. One output neuron was defined, and several input neurons were connected to it by regular connections or through defined dendrites. Initially all W_i_ were set to their initial value, as stated for each simulation.

#### Stimulations

We simultaneously stimulated above-threshold the input neurons at 10 Hz.

#### Learning Rule for synaptic learning

For every pair of a sub-threshold stimulation and an evoked spike (originated from two different input neurons), the connections weights were modulated according to the following equation:

2 $$\delta W=0.05\ast \exp (\frac{-|{\rm{\Delta }}|}{15})\ast sign({\rm{\Delta }})$$

were ${\rm{\delta }}{\rm{W}}$ is the change in the weight, W_i_, and $\triangle$ measured in milliseconds is the time-lag between the sub threshold stimulation and evoked spike. The learning curve had a cutoff at 50 ms. The weights were updated the following way: ${{\rm{W}}}_{{\rm{i}}}:={{\rm{W}}}_{{\rm{i}}}(1+\delta W)$ and the minimum possible weight value was set to 0.001.

#### Learning Rule for dendritic learning

For every pair of a sub-threshold stimulation and an evoked spike originated from two different dendrites, the dendritic weights, W_Di_, were modulated according to the same above-mentioned equation as for synaptic learning, where ${\rm{\delta }}{\rm{W}}$ stands for the change in the dendritic weight: ${{\rm{W}}}_{{\rm{Di}}}:={{\rm{W}}}_{{\rm{Di}}}(1+\delta W)$.

### *In-Vitro* Experiments

For experimental methods see ref.^26^. Additional procedures are detailed below:

#### Extracellular threshold estimation

The extracellular threshold remained stable during the experiments. After the training of coupled intra- and extra- stimulations, the extracellular threshold was re-estimated every several minutes in order to validate the stability of the parameters used in the experiment. The learning effect is visible only after several minutes, typically 3–6 minutes. The results presented in Fig. 4 are recorded ~5 minutes after training.

#### Experiments protocol

An extracellular electrode was selected and both intra- and extra- cellular thresholds were estimated. The neuronal response latency, NRL, and its stability for the extracellular electrode were estimated in order to accurately adjust the arrival timings of the intra- and extra- cellular stimulations. In order to enhance possible adaptation, the time-lags between stimulations were set to 2–5 ms (following the expected learning curve), and sub-threshold stimulation amplitudes close to the threshold (with visible local depolarization). We note that an above-threshold extracellular stimulation given shortly, e.g. 2 ms, after the intracellular stimulation, does not result in an evoked spike, and can be used to enhance the adaptation. The thresholds and NRL were rechecked at the end of the experiment, in order to ensure its stability.

#### Statistical analysis

The demonstrated results were quantitatively repeated tens of times on many cultures. In particular, the effect of Fig. 4e,f was observed in more than 85% of such examined experiments, where Δ was in the range of 2–5 ms. The increase in the local depolarization (Fig. 4e) typically ranges between 100–300% and the enhance from small local depolarization to evoked spikes recorded intracellularly was extended to one or several lowered stimulation amplitudes. A reverse learning procedure (Fig. 5) was also observed tens of times on many cultures and resulted in more than 90% with no effect or weakening of the local depolarization.

## Electronic supplementary material

Supplementary information
