# Supplementary material for: Adaptive nodes enrich nonlinear cooperative learning beyond traditional adaptation by links
Source: Sci Rep. 2018 Mar 23;8:5100. doi: 10.1038/s41598-018-23471-7 (PMC5865176; doi:10.1038/s41598-018-23471-7)
Supplement: Supplementary file 1 — Supplementary information [file 41598_2018_23471_MOESM1_ESM.pdf]

Adaptive nodes enrich nonlinear cooperative learning beyond traditional adaptation by links

Shira Sardi[1], Roni Vardi[1,2], Amir Goldental[1], Anton Sheinin[3], Herut Uzan[1] and Ido Kanter[1,2,*]

[1]Department of Physics, Bar-Ilan University, Ramat-Gan 52900, Israel
[2]Gonda Interdisciplinary Brain Research Center and the Goodman Faculty of Life Sciences, Bar-Ilan University, Ramat-Gan 52900, Israel
[3]Sagol School of Neuroscience, Tel Aviv University, Tel Aviv, Israel

[*]e-mail: ido.kanter@biu.ac.il

Supplemental Figures

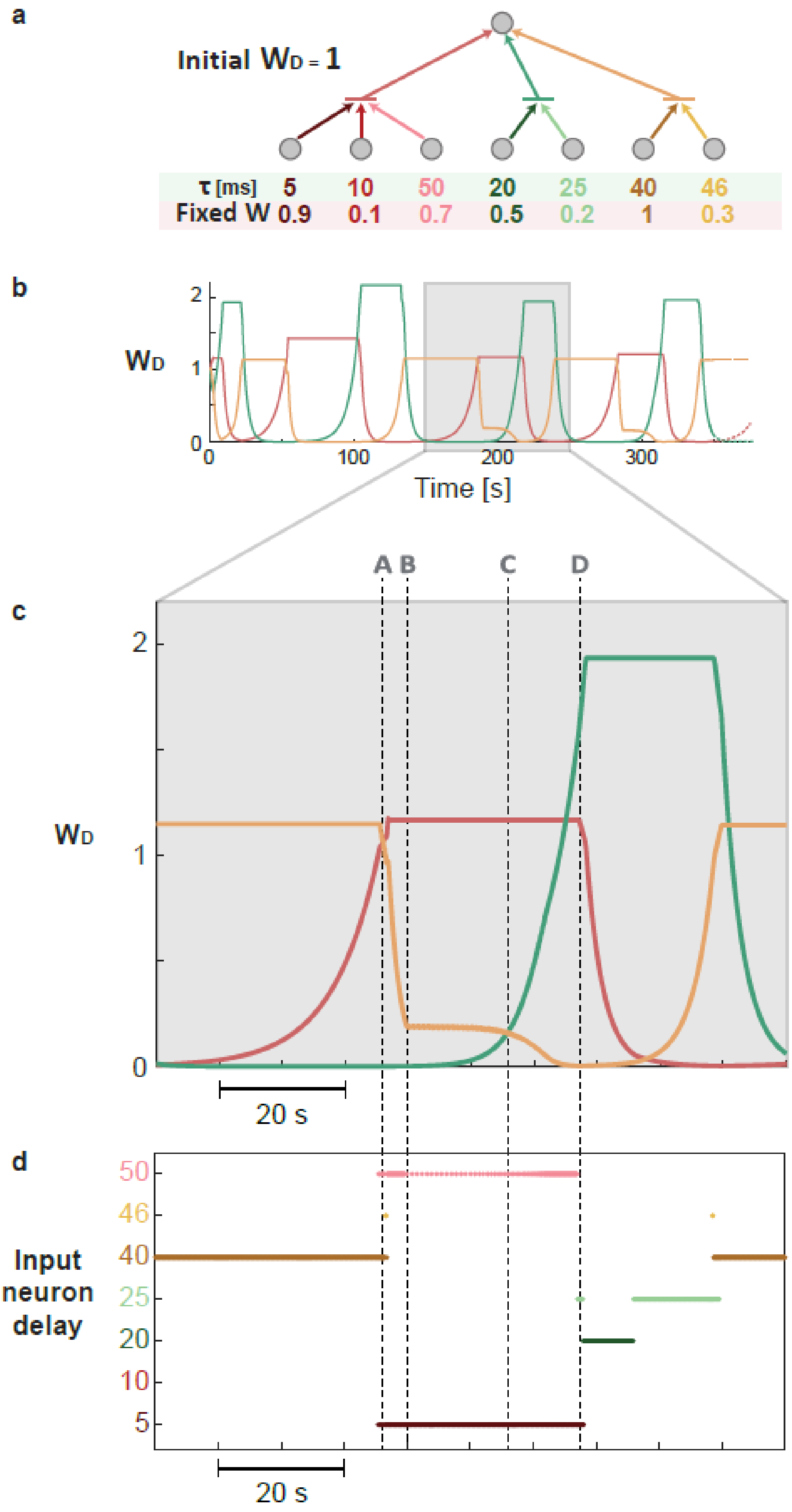
a
Initial W_D = 1
τ [ms] 5 10 50 20 25 40 46
Fixed W 0.9 0.1 0.7 0.5 0.2 1 0.3
b
W_D
Time [s]
c
A B C D
W_D
20 s
d
Input neuron delay
50
46
40
25
20
10
5
20 s

Supplementary Figure 1| Zoom in on the dynamics of the dendrite weights in Fig. 3d$_1$ simulations. a, A schema of a perceptron with seven inputs and delays (w, τ, color coded) as in Fig. **3b**. **b,** Same as in Fig. **3d$_1$**. **c,** Zoom-in on the grey area in **b**. **d,** The output neuron spike scheduling for the time slot in **c**, color coded following the delays of the weights generating the evoked spike.

Four steps of the dynamics are presented (A-D, dashed black lines):

Step A: The input neuron with 50 ms delay starts to generate evoked spikes, in addition to the spikes after 5 ms. The orange-dendrite weakens rapidly as a result of its 46 ms sub-threshold stimulations, arriving only 4 ms prior to the new evoked spikes. The steep weakening of the orange-dendrites continues as long as the 50 ms delay generates evoked spikes at high rates, between steps A and B.

Step B: The input neuron with 50 ms delay generates now spikes at much lower rates, which moderate the weakening of the orang-dendrite. The origin of this slowdown in the firing rates is its effective weight ~0.7*1.2=0.84, which is below the threshold 1 (the weight strength is 0.7 (**a**) and its red-dendrite strength is ~1.2 (**c**)). Generating evoked spikes via the 50 ms weight requires temporarily high membrane potential in comparison to the resting membrane potential. This condition is achieved as a result of the termination of prior evoked spikes after 40 ms and after 46 ms. These input neurons now generate depolarization after 40 and 46 ms which increases the temporarily membrane potential, until around step B.

Step C: The strengthening of the green-dendrite after step B generates sub-threshold stimulations at 20 ms and 25 ms, which increase the membrane potential after 50 ms and generate low rates of evoked spikes after 50 ms. The strengthening of the green-dendrites is enhanced while approaching step C and accordingly the firing rates of the 50 ms delay. Now the orange-dendrite weakens rapidly again, as in Step A.

Step D: The green-dendrite generates evoked spikes via the 20 ms and 25 ms delays. Now the red-dendrite weakens as a result of its sub-threshold stimulations after 5 ms and 10 ms.

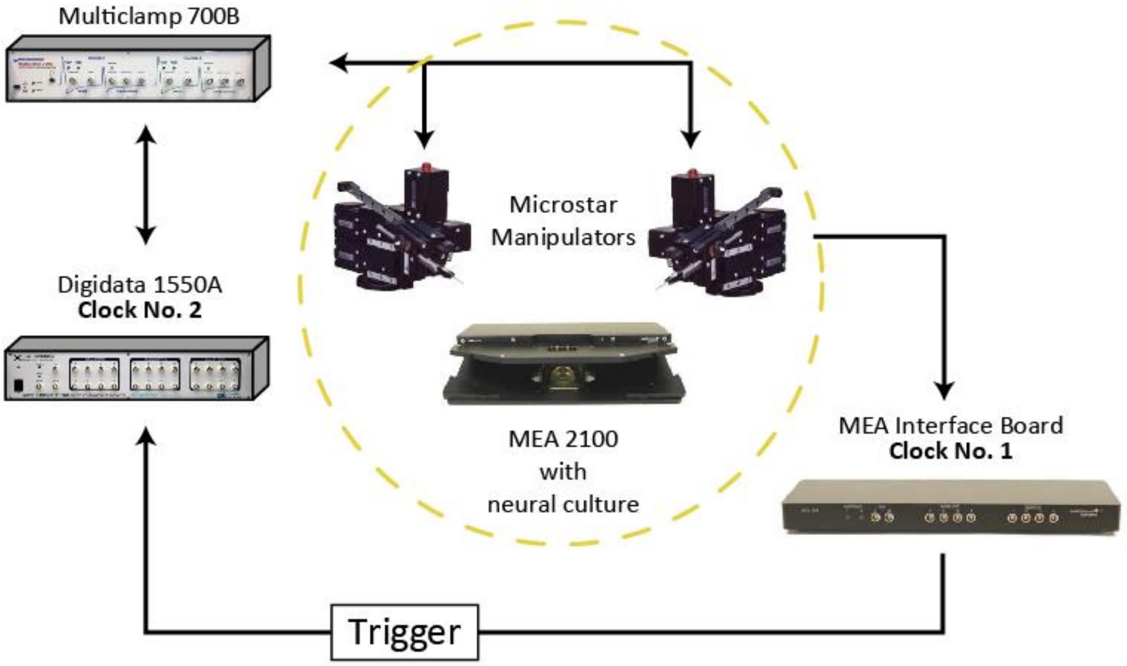

Supplementary Figure 2| A schema of the experimental setup combining multi-electrode array and patch clamp recordings. The multi-electrode array, MEA 2100, is controlled by the MEA interface board and a computer. The Patch clamp sub-system consists of several microstar manipulators. Stimulations and recordings are implemented using multiclamp 700B and Digidata 1550A and are controlled by a different computer. The time of the MEA system is controlled by a clock placed in the MEA interface board (clock No. 1), and the time of the patch subsystem is controlled by a clock placed in the Digidata 1550A (clock No. 2). The relative timings are controlled by triggers sent from the MEA interface board to the Digidata, using leader-laggard configuration.
